# Cinnamon-Derived Phytonutrients as Modulators of Ion Channels and G Protein-Coupled Receptor Signaling in Metabolic Diseases

**DOI:** 10.3390/nu18030547

**Published:** 2026-02-06

**Authors:** Raymond Rubianto Tjandrawinata, Bayu Perkasa Rosari, Rony Abdi Syahputra, Reggie Surya, Fahrul Nurkolis

**Affiliations:** 1 School of Bioscience, Innovation and Technology, Atma Jaya Catholic University of Indonesia, Jakarta 12930, Indonesia; 2 Dexa Laboratories of Biomolecular Sciences, Dexa Medica, Industri Selatan V PP-7, Jababeka 2, Cikarang 17550, Indonesia; 3 Department of Anatomical Pathology, School of Medicine and Health Sciences, Atma Jaya Catholic University of Indonesia, Jakarta 14440, Indonesia; 4 Department of Pharmacology, Faculty of Pharmacy, Universitas Sumatera Utara, Medan 20155, Indonesia; 5 Department of Food Technology, Faculty of Engineering, Bina Nusantara University, Jakarta 11480, Indonesia; 6 Master of Basic Medical Science, Faculty of Medicine, Universitas Airlangga, Surabaya 60131, Indonesia; fahrul.nurkolis.mail@gmail.com; 7 Medical Research Center of Indonesia, Surabaya 60281, Indonesia; 8 Institute for Research and Community Service, State Islamic University of Sunan Kalijaga (UIN Sunan Kalijaga), Yogyakarta 55281, Indonesia

**Keywords:** cinnamon, nutraceuticals, ion channels, GPCR signaling, TRP channels, calcium signaling, metabolic diseases, insulin secretion, energy metabolism, precision nutrition

## Abstract

Metabolic diseases such as type 2 diabetes and obesity are increasingly recognized as disorders of dysregulated cellular communication rather than solely enzymatic or transcriptional dysfunction. While conventional therapies primarily target metabolic enzymes and nuclear receptors, growing evidence highlights ion channels and G protein-coupled receptors (GPCRs) at the cell membrane as critical upstream regulators of glucose homeostasis, energy expenditure, and inflammation. Cinnamon (*Cinnamomum* spp.), a widely consumed nutraceutical, has demonstrated consistent antidiabetic and antiobesity effects; however, its actions at the membrane signaling interface remain underappreciated. This review synthesizes emerging evidence that cinnamon-derived phytonutrients, particularly cinnamaldehyde, eugenol, and polyphenolic compounds, modulate key ion channels and GPCR pathways involved in metabolic regulation. We discuss how cinnamon influences calcium signaling, transient receptor potential (TRP) channels, and metabolite- and hormone-sensing GPCRs, thereby affecting insulin secretion, incretin release, appetite control, thermogenesis, and inflammatory tone. A central highlight of this review is the crosstalk between ion channels and GPCRs in metabolic tissues, illustrating a systems-level mechanism through which cinnamon exerts pleiotropic metabolic benefits. Translational implications are explored, including the potential of cinnamon to complement existing antidiabetic therapies and its relevance within precision nutrition frameworks. By focusing on the cell membrane as an integrative signaling hub, this review reframes cinnamon as a membrane-active nutraceutical capable of restoring metabolic balance through coordinated modulation of ion channel GPCR networks.

## 1. Introduction

Metabolic diseases such as type 2 diabetes and obesity are fundamentally disorders of dysregulated cellular signaling in metabolic tissues. In metabolic disease states, however, key signaling pathways become imbalanced, leading to chronic hyperglycemia, insulin resistance, dyslipidemia, and inflammation [1,2,3]. Traditional therapeutic strategies have predominantly targeted enzymes (e.g., α-glucosidase in the gut, dipeptidyl peptidase-4 (DPP-4) in circulation) or gene regulators (e.g., nuclear receptors like PPARγ, or energy sensors like AMPK) to improve metabolic control. While such enzyme- and gene-centric therapies (e.g., acarbose, DPP-4 inhibitors, thiazolidinediones, metformin) can be effective, they often address downstream metabolic processes and may not fully restore the complex signaling balance in cells. Emerging evidence suggests that membrane signaling mechanisms, in particular, ion channels and G protein-coupled receptors (GPCRs) on the cell surface, serve as critical upstream regulators of metabolic functions. By acting as molecular sensors and transducers on cell membranes, ion channels, and GPCRs, integrate hormonal, nutritional, and neuronal signals to orchestrate metabolic responses in real time [1,4,5]. This has led to growing interest in leveraging these membrane proteins as therapeutic targets for metabolic diseases.

Notably, some nutraceuticals and dietary bioactives appear to exert metabolic benefits by modulating membrane signaling pathways, beyond their classical antioxidant or enzyme-inhibitory properties [6]. Cinnamon (*Cinnamomum* species) is a prime example of a functional food with reported antidiabetic and antiobesity effects, yet the underlying mechanisms remain only partially understood. Recent studies have begun to uncover that cinnamon’s active phytochemicals can influence ion channel activity and GPCR signaling in key metabolic tissues, thereby modifying insulin secretion, inflammation, and energy expenditure [7,8]. This review will comprehensively examine how cinnamon-derived phytonutrients modulate membrane-associated targets, specifically ion channels and GPCRs, and the implications of these actions for managing metabolic diseases.

**Figure 1:**
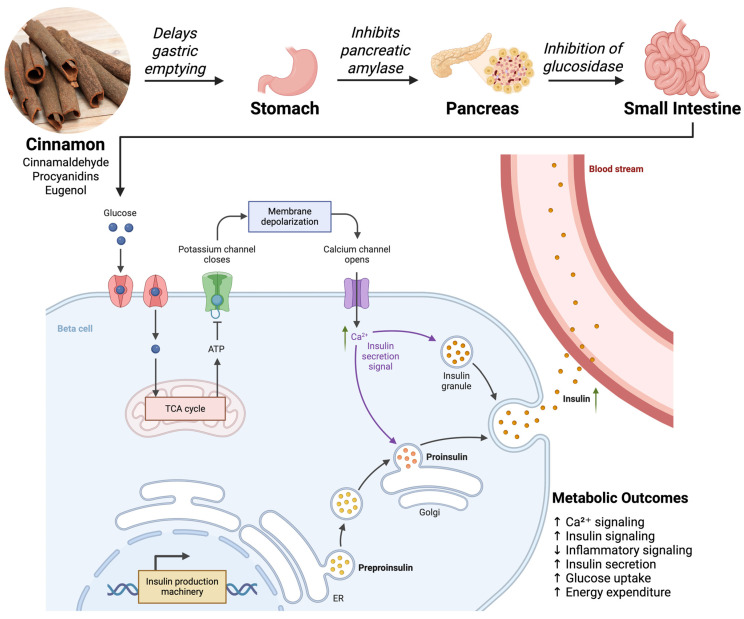
Cinnamon as a membrane-active nutraceutical in metabolic regulation. Cinnamon-derived phytonutrients interact with ion channels and GPCRs at the cell membrane, initiating rapid signaling events that regulate insulin secretion, incretin release, energy expenditure, and inflammatory tone. Figure created via Biorender Premium License by Fahrul Nurkolis. ↑ Increase; ↓ decrease.

Therefore, this review aims to systematically synthesize and critically evaluate emerging evidence on how cinnamon-derived phytonutrients modulate ion channels and GPCR signaling pathways involved in metabolic regulation. Specifically, this article focuses on membrane-centered mechanisms underlying cinnamon’s effects on calcium signaling, transient receptor potential (TRP) channels, and key metabolite- and hormone-sensing GPCRs across metabolic tissues, including the pancreas, gut, adipose tissue, skeletal muscle, and the neuroendocrine axis (Figure 1). By integrating molecular, cellular, physiological, and translational findings, this review seeks to elucidate how membrane signaling serves as an upstream regulatory hub linking cinnamon intake to improvements in insulin secretion, energy expenditure, appetite control, and inflammatory tone.

The novelty of this review lies in reframing cinnamon not merely as an antioxidant or enzyme-modulating nutraceutical, but as a membrane-active signaling modulator that operates through coordinated ion channel–GPCR networks. Unlike previous reviews that primarily emphasize cinnamon’s effects on insulin sensitivity, glycemic indices, or transcriptional regulators, this work highlights the underexplored yet critical role of membrane excitability, sensory signaling, and receptor crosstalk in mediating cinnamon’s pleiotropic metabolic benefits. Furthermore, by emphasizing ion channel–GPCR crosstalk as a systems-level mechanism, this review introduces a conceptual framework in which cinnamon exemplifies “network nutraceutical pharmacology,” bridging sensory biology, endocrinology, and immunometabolism.

### Search Strategy and Study Selection

A comprehensive literature search was conducted to identify relevant studies investigating cinnamon, its phytochemical constituents, and their interactions with ion channels, GPCRs, and metabolic signaling pathways. Electronic databases, including PubMed/MEDLINE, Scopus, and Web of Science, were searched from inception to the most recent available date (January 2015 to December 2025). Search terms were constructed using combinations of keywords and Boolean operators, including: “cinnamon” OR “Cinnamomum”, “nutraceutical”, “ion channel”, “calcium signaling”, “TRP channels”, “G protein-coupled receptor” OR “GPCR”, “metabolism”, “insulin signaling”, “glucose homeostasis”, and “type 2 diabetes”. Reference lists of selected articles were also manually screened to identify additional relevant studies. Study selection followed a two-step process. First, titles and abstracts were screened to exclude irrelevant articles, conference abstracts without full text, and non-English publications. Second, full-text articles were assessed for eligibility. We included in vitro, in vivo, and human studies, as well as high-quality mechanistic and translational reviews, provided they offered direct or indirect evidence linking cinnamon or its bioactive compounds to ion channel or GPCR-mediated metabolic regulation. Studies focusing solely on antioxidant or anti-inflammatory effects without relevance to membrane signaling were excluded.

## 2. Nutraceuticals and Membrane Signaling in Metabolic Diseases

While nutraceuticals like polyphenols and spices are often recognized for antioxidant activity or improvement of glycemic indices, their bioactivity extends far beyond these roles. Emerging research reveals that many dietary compounds can modulate cell membrane receptors and ion channels, effectively acting as signaling molecules [6,9,10]. The cell membrane functions as a dynamic sensing platform in metabolic regulation, hosting numerous receptors and channels that detect extracellular nutrients, hormones, and even the components of our diet [1,7]. Nutraceutical compounds can interact with these membrane proteins to trigger or modulate signaling cascades, influencing metabolism in ways unrelated to direct enzyme inhibition.

Cinnamon is a particularly potent nutraceutical in this context. Beyond improving insulin resistance and oxidative stress markers, cinnamon has been shown to modulate calcium influx and vascular tone, indicating an action on ion channels in smooth muscle [6,11,12]. It also influences the release of gut hormones like glucagon-like peptide-1 (GLP-1) and can reduce gastric emptying and appetite via sensory pathways [7,13]. These findings suggest that the bioactives in cinnamon target membrane-bound sensors that regulate metabolism.

In metabolic homeostasis, ion channels and GPCRs on cell membranes are pivotal for sensing and responding to nutritional status. Notable examples include pancreatic β-cell ion channels that regulate insulin secretion, and GPCRs in the gut and adipose tissue that respond to metabolites [14,15]. In the context of glucose homeostasis, voltage-gated Ca^2+^ channels in pancreatic β-cells mediate Ca^2+^ influx that triggers insulin exocytosis, and inhibition of these channels abolishes glucose-stimulated insulin release [16]. In parallel, GPCRs such as the glucagon-like peptide-1 (GLP-1) receptor on β-cells amplify insulin secretion in response to incretin hormones [1].

Lipid metabolism is likewise regulated by membrane signaling mechanisms in adipose tissue. GPCRs on adipocytes, including β-adrenergic receptors, stimulate lipolysis and fatty acid oxidation, while specific ion channels influence adipocyte differentiation and lipid storage. In brown and beige adipose tissue, activation of β_3_-adrenergic receptors promotes thermogenic fat burning [1,17]. Consistent with this framework, cinnamon supplementation has been associated with increased fat oxidation in human studies, potentially through activation of transient receptor potential (TRP) ion channels that engage sympathetic β-adrenergic pathways [18].

Transient receptor potential (TRP) ion channels expressed in sensory nerves and metabolic tissues act as molecular thermometers and nutrient sensors, affecting feeding behavior and energy expenditure [7,19]. GPCRs like GPR120 (the omega-3 fatty acid receptor) regulate appetite and energy partitioning by coordinating signals between the gut, adipose tissue, and brain [1,20]. Cinnamon’s pungent compound cinnamaldehyde, for example, activates the TRPA1 ion channel, which has been linked to enhanced thermogenesis and reduced food intake via gut–brain signaling [7].

By engaging these membrane signaling systems, nutraceuticals can produce integrated metabolic effects. In the case of cinnamon, its constituents appear to mimic hormonal signals or sensory stimuli: triggering insulin release like incretin hormones, stimulating thermogenesis like cold exposure or capsaicin, and modulating appetite via gut peptides and neuronal pathways. Rather than acting solely as calorie-independent insulin sensitizers or antioxidant agents, compounds such as those in cinnamon actively engage the body’s own cellular communication networks to restore metabolic balance [7,21].

## 3. Why Ion Channels and GPCRs Are Underrated Targets in Metabolic Diseases

Despite their importance, ion channels and GPCRs have historically been under-targeted in metabolic disease therapy compared to enzymes and nuclear receptors. One reason is the dominance of enzyme-centric drugs: for example, inhibition of digestive enzymes (α-glucosidase inhibitors) to reduce glucose absorption, or inhibition of DPP-4 enzyme to prolong incretin action, have been successful strategies in type 2 diabetes [22]. Similarly, gene-level targets like PPARγ (activated by thiazolidinediones) and AMPK (activated indirectly by metformin) address metabolic defects by altering transcription or cellular energy state [23]. These approaches, however, often tackle downstream effects and may not fully address acute dysregulation in intercellular signaling. Ion channels and GPCRs, in contrast, serve as immediate regulators of cell function and inter-tissue communication, but their therapeutic exploitation in metabolism has been limited by complexity and side effect concerns [24].

Ion channels and GPCRs play essential roles in insulin secretion, adipose tissue function, inflammation, and energy expenditure. In pancreatic β-cells, insulin secretion is tightly controlled by membrane excitability. Closure of ATP-sensitive K^+^ (K-ATP) channels in response to increased ATP from glucose metabolism depolarizes the membrane and opens voltage-gated Ca^2+^ channels, leading to insulin exocytosis [16]. Sulfonylureas such as glibenclamide exploit this mechanism therapeutically, and recent evidence indicates that glibenclamide also agonizes the TRPA1 ion channel, suggesting previously unrecognized interactions with sensory ion channels [19]. Cinnamon-derived cinnamaldehyde can activate TRPA1 directly in β-cells, inducing Ca^2+^ influx and basal insulin release independently of K-ATP channel closure, primarily demonstrated in preclinical models, thereby offering a complementary route for enhancing insulin secretion [7].

Membrane signaling is equally critical in adipose tissue differentiation and metabolism. Specific ion channels, including TRPV4, regulate adipocyte function by influencing adipogenesis, oxidative metabolism, and inflammatory signaling; activation of TRPV4 has been shown to suppress browning of white adipose tissue and promote insulin resistance in preclinical models [25,26]. Conversely, GPCRs such as GPR120 expressed on adipocytes and macrophages mediate fatty acid-induced anti-inflammatory and insulin-sensitizing effects [20]. Because these membrane regulators are largely overlooked by mainstream metabolic drugs, they represent underexplored targets. In this context, cinnamon polyphenols have been reported to enhance adipocyte glucose uptake and inhibit adipogenesis through pathways involving AMPK activation and suppression of pro-adipogenic gene expression, potentially acting upstream at the level of GPCRs or ion channels [7].

Ion channels and GPCRs also integrate inflammatory and thermogenic pathways that are central to metabolic disease progression. Chronic low-grade inflammation in adipose and hepatic tissues contributes to insulin resistance and is regulated in part by Ca^2+^-permeable ion channels and GPCR-mediated immune signaling. Activation of GPCRs such as GPR120 by omega-3 fatty acids promotes anti-inflammatory macrophage phenotypes and improves insulin sensitivity [20]. In parallel, β-adrenergic GPCRs in brown and beige adipocytes drive thermogenesis by upregulating uncoupling protein 1 (UCP1) and increasing energy expenditure [1]. Although most pharmacological treatments do not directly target these pathways, nutraceuticals may provide indirect modulation. Cinnamaldehyde has been shown to induce UCP1 expression and fatty acid oxidation genes in adipose tissue, consistent with activation of sympathetic and β-adrenergic thermogenic signaling [7]. Cinnamon has also demonstrated anti-inflammatory effects in metabolic tissues, potentially via GPCR-mediated suppression of NF-κB signaling and pro-inflammatory cytokine release [27].

In summary, ion channels and GPCRs are integral regulators of insulin release, adipose metabolism, and inflammatory energy balance, yet they remain underutilized targets in treating metabolic syndrome and diabetes. This underrating stems from the historical success of enzyme inhibitors and the complexity of membrane signaling (many ion channels and GPCRs are ubiquitously expressed, raising concern for off-target effects). However, growing evidence of their specificity in metabolic organs argues for revisiting these targets. Nutritional and pharmacological modulation of ion channels (like TRP channels, K^+^ and Ca^2+^ channels) and GPCRs (adrenergic receptors, free fatty acid receptors, etc.) can produce multifaceted metabolic improvements [1,19]. The challenge and opportunity lie in harnessing these membrane proteins with precision, possibly through multi-component nutraceuticals like cinnamon, which may achieve a balanced modulation of pathways due to its “polypharmacology” across different targets.

## 4. Phytochemistry of Cinnamon Relevant to Membrane Targets

**Figure 2:**
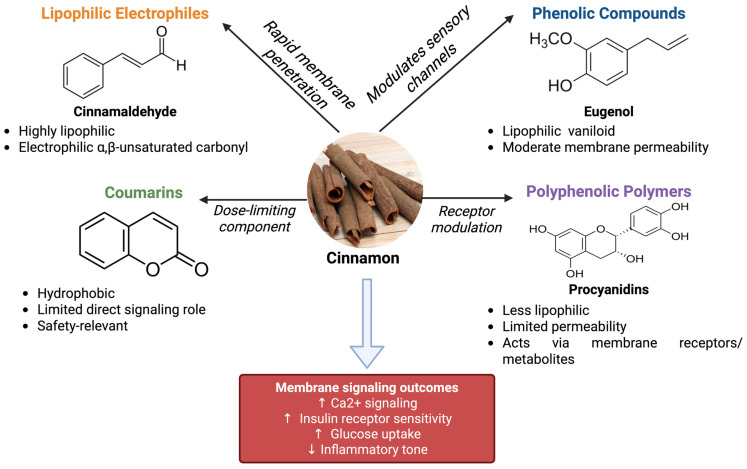
Major cinnamon phytochemicals are involved in membrane signaling. Cinnamon contains lipophilic electrophiles (e.g., cinnamaldehyde), phenolics (e.g., eugenol), and polyphenolic polymers that differ in membrane permeability and signaling properties, enabling coordinated modulation of ion channels and GPCRs relevant to metabolic regulation. This figure was created via Biorender Premium License by Fahrul Nurkolis. ↑ Increase; ↓ decrease.

The genus *Cinnamomum* encompasses several species used as the spice “cinnamon,” with *Cinnamomum verum* (“Ceylon cinnamon”) and *C. cassia* (“Chinese cinnamon”) being the most common. The phytochemical profile of cinnamon bark includes a variety of bioactive compounds, of which a subset is particularly relevant to modulating membrane proteins such as ion channels and GPCRs. Key cinnamon-derived phytonutrients include cinnamaldehyde, a trans-α,β-unsaturated aldehyde that gives cinnamon its distinctive aroma and flavor (Figure 2). Cinnamaldehyde constitutes 60–80% of the essential oil of cinnamon bark [27]. It is a highly lipophilic molecule capable of penetrating cell membranes, and it contains an electrophilic α,β-unsaturated carbonyl group that can form covalent adducts with cysteine residues on proteins. This chemical reactivity underlies cinnamaldehyde’s potent biological actions as an agonist of TRPA1 ion channels. Its electrophilic nature allows it to activate sensor proteins like TRPA1 by covalently binding to cysteine sites on the channel, triggering Ca^2+^ influx in cells [7,28]. This mechanism is analogous to other pungent electrophiles (allyl isothiocyanate from mustard, allicin from garlic) that also activate TRP channels [19]. In addition, cinnamaldehyde can be metabolized in vivo to cinnamic acid, which we will discuss as a modulator of certain GPCRs (notably GPR109A) [7].

Another phytonutrient is eugenol, a phenolic aromatic compound (4-allyl-2-methoxyphenol) found in smaller quantities in cinnamon bark and in higher concentrations in cinnamon leaf oil and clove oil (Figure 2). Chemically, eugenol is a vanilloid and can interact with membrane channels; it has been shown to activate or modulate TRP channels, though at higher concentrations than canonical agonists like capsaicin [29,30]. For instance, eugenol can activate TRPV1 (the capsaicin receptor) in sensory neurons, albeit less potently than capsaicin [29]. It can also activate TRPA1 channels in trigeminal neurons [31]. Through these interactions, eugenol might influence metabolic processes by affecting sensory nerve signaling (e.g., altering pain/inflammation circuits) and muscle metabolism. A recent study even suggested eugenol can mimic some effects of exercise on muscle by activating TRPV1-mediated Ca^2+^-calcineurin signaling, promoting oxidative muscle fibers [32]. Thus, eugenol’s lipophilicity and ability to cross membranes allow it to reach and modulate ion channels involved in metabolic regulation (though its role in metabolic disease specifically is less documented than cinnamaldehyde’s).

Cinnamon, especially *C. cassia*, contains coumarin (1,2-benzopyrone) and related compounds (Figure 2). Coumarin is responsible for the sweet aroma of cassia cinnamon, but it is also a hepatotoxic agent at high doses. From a membrane pharmacology perspective, coumarins are planar, hydrophobic molecules that can intercalate into lipid bilayers and potentially modulate membrane-bound enzymes or receptors. Coumarin itself is not known to directly activate any ion channel or GPCR relevant to metabolism; however, it does bear structural similarity to certain flavonoids and can influence signaling indirectly. Importantly, coumarin’s safety profile constrains cinnamon dosing; prolonged high intake of cassia cinnamon can lead to liver damage due to coumarin accumulation. Tolerable daily intake of coumarin is estimated at ~0.1 mg per kg body weight [27,33,34]. Therefore, while coumarins may not be major effectors of membrane signaling for metabolic benefits, they are relevant when considering nutraceutical dosing and safety (addressed in Section 8.2).

Cinnamon bark contains water-soluble polyphenolic polymers, including procyanidins (type A proanthocyanidins) and catechins. A specific polyphenol termed MHCP (methyl hydroxychalcone polymer) was isolated from cinnamon and reported to exhibit insulin-mimetic activity in adipocytes. These polyphenols can activate insulin receptor autophosphorylation and glucose uptake in cells, potentially through affecting the cell surface insulin receptor or downstream signaling. Polyphenols can also bind to cell membranes or membrane proteins, altering their conformation or clustering. For example, procyanidin oligomers might interact with lipid rafts and influence insulin receptor localization, thereby enhancing insulin signaling sensitivity [12,27]. In the context of ion channels and GPCRs, polyphenols can act as allosteric modulators or bias receptor signaling. Cinnamon’s procyanidins have been shown to increase GLUT4 translocation in muscle and fat cells and regulate enzymes in gluconeogenesis, implying an upstream receptor-mediated effect (possibly through GPCRs like the insulin receptor or others) [19,35]. While these large polyphenols are less lipophilic than cinnamaldehyde, their metabolites (after gut microbiota processing) may become bioactive and capable of crossing membranes to engage GPCRs such as GPR109A (which responds to certain phenolic acids) [7,36]. The bioavailability of cinnamon polyphenols is relatively low, but even their presence in the gut can modulate gut membrane receptors and channels, influencing GLP-1 secretion and glucose absorption.

The membrane interactions of cinnamon’s phytochemicals are governed by their lipophilicity. Cinnamaldehyde and eugenol are highly lipophilic and readily diffuse across cell membranes, enabling direct access to hydrophobic pockets on ion channels/GPCRs. They can also embed in the lipid bilayer and alter membrane fluidity, which might indirectly affect membrane protein conformation [37,38]. Polyphenols, being more polar, may interact with membrane surface receptors or require transport mechanisms or metabolism to gain entry into cells. The blend of both lipophilic and hydrophilic compounds in cinnamon might be synergistic; for instance, cinnamaldehyde might trigger acute ion channel responses, while polyphenols sustain receptor signaling or insulin-sensitizing actions over longer periods [39,40,41]. Understanding these phytochemical properties is crucial for appreciating how cinnamon extracts reach and modulate membrane targets.

In summary, cinnamon provides a phytochemical cocktail where cinnamaldehyde and eugenol act as rapid membrane-penetrating agonists of channels (like TRPA1/TRPV1), coumarins contribute to effects (and safety considerations) at the membrane level, and polyphenolic polymers enhance receptor-mediated signaling for insulin and metabolic control. These compounds’ varying structures and lipophilicities allow them to collectively influence a broad array of membrane-associated targets, laying the foundation for cinnamon’s multifaceted role in metabolic disease modulation.

## 5. Ion Channel Modulation by Cinnamon-Derived Compounds

**Figure 3:**
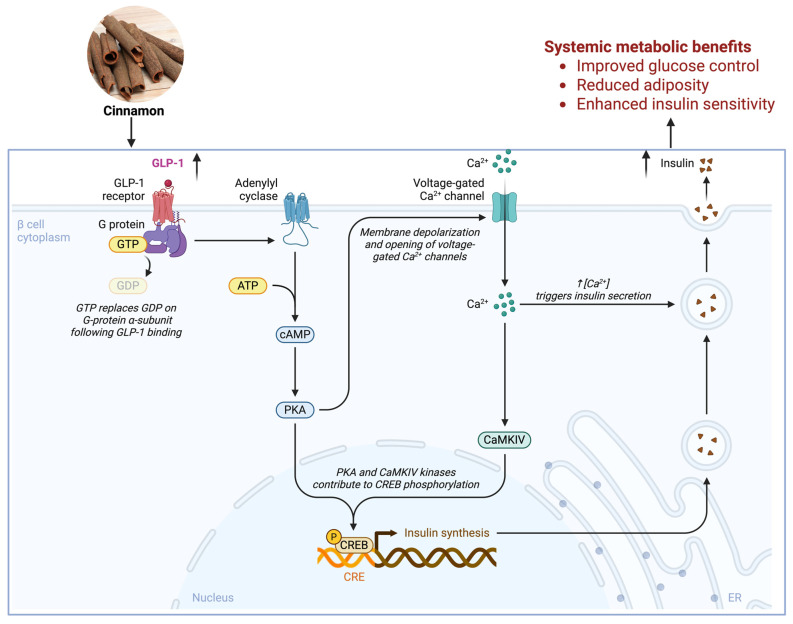
Cinnamon-mediated ion channel signaling in metabolic tissues. Activation of TRP channels by cinnamon phytonutrients induces Ca^2+^ influx across metabolic and sensory cells, promoting insulin secretion, incretin release, appetite regulation, and energy expenditure. Figure created via Biorender Premium License by Fahrul Nurkolis.

Ion channels are pore-forming membrane proteins that enable ions (Ca^2+^, K^+^, Na^+^, etc.) to cross cell membranes, thereby controlling electrical excitability, signal transduction, and intracellular ionic milieu. In metabolic tissues, ion channels translate metabolic cues into functional responses. For example, in pancreatic β-cells, changes in ATP/ADP ratio regulate ion channels to trigger insulin release [16,42]. Cinnamon-derived compounds have shown the ability to modulate several classes of ion channels that are relevant to metabolic regulation (Figure 3). This section focuses on how cinnamon phytonutrients affect ion channel signaling, with emphasis on calcium channels and the TRP (transient receptor potential) family, and how these actions influence metabolic outcomes.

### 5.1. Calcium (Ca^2+^) Signaling in Metabolic Regulation

Calcium ions (Ca^2+^) serve as a universal second messenger in cells, and calcium signaling is particularly crucial in metabolic regulation. In pancreatic β-cells, the rise in intracellular Ca^2+^ is the direct trigger for insulin granule exocytosis. Normally, glucose uptake leads to an increased ATP/ADP ratio that closes K-ATP channels, depolarizing the cell membrane and opening voltage-dependent Ca^2+^ channels (primarily L-type Ca^2+^ channels). The resulting Ca^2+^ influx raises cytosolic [Ca^2+^]_i, causing insulin vesicles to fuse with the membrane and release insulin [16,43,44].

Cinnamon compounds appear to modulate Ca^2+^ signaling pathways in both direct and indirect ways. Cinnamaldehyde, as a TRPA1 agonist, indirectly raises intracellular Ca^2+^ in β-cells by opening the TRPA1 cation channel (which is Ca^2+^-permeable) on the plasma membrane. Indeed, TRPA1 has been detected in rodent β-cells, and its activation by cinnamaldehyde led to basal insulin release even without high glucose, due to Ca^2+^ influx through the TRPA1 channel [7]. This suggests cinnamon can augment insulin secretion beyond the glucose-triggered pathway, potentially useful for improving β-cell function in diabetes. Moreover, cinnamon’s effect on Ca^2+^ channels in β-cells may enhance first-phase insulin release. One study noted that cinnamon extract improved β-cell responsiveness and suggested it upregulated genes for calcium handling, although detailed mechanisms were not fully delineated [12,19].

Beyond the pancreas, Ca^2+^ channels in muscle and liver cells influence metabolic enzyme activity and gene expression (e.g., Ca^2+^/calmodulin-dependent kinase signaling affects glycogen metabolism). While not documented as extensively, cinnamon polyphenols might modulate these Ca^2+^ signaling cascades indirectly via improving mitochondrial function or redox state, thus maintaining Ca^2+^ homeostasis [45,46].

In the cardiovascular system, which is often affected in metabolic syndrome, cinnamon has been shown to help maintain normal vascular contractility by modulating Ca^2+^ influx in smooth muscle. This could indicate a mild calcium-channel blocking effect of certain cinnamon compounds (possibly polyphenols or coumarin), leading to vasodilation and blood pressure reduction. Indeed, an umbrella review of RCTs found cinnamon supplementation associated with blood pressure improvements and posited a mechanism involving calcium channel modulation and increased nitric oxide (NO) bioavailability [6,11].

Another aspect of Ca^2+^ signaling in metabolism is ER stress and insulin resistance. Excess intracellular Ca^2+^ or dysregulated Ca^2+^ release from the endoplasmic reticulum (ER) can trigger stress pathways that impair insulin signaling. While direct evidence is sparse, it is conceivable that cinnamon’s antioxidants help buffer Ca^2+^ oscillations, thereby reducing ER stress in insulin-resistant cells. By preventing Ca^2+^ overload, cinnamon could protect insulin receptor signaling and improve glucose uptake [12,47].

In summary, cinnamon influences Ca^2+^-dependent processes critical for metabolism. *In β-cells*, it can enhance insulin secretion via Ca^2+^ influx (through TRPA1 activation and possibly through better voltage-Ca^2+^ channel function). In vessels, it aids Ca^2+^ control, contributing to blood flow and nutrient delivery, and at the cellular level, it might mitigate Ca^2+^-related stress that leads to insulin resistance [6,7,18]. These multi-faceted effects on calcium signaling illustrate how a nutraceutical can target an elemental signaling ion to achieve metabolic benefits.

### 5.2. Transient Receptor Potential (TRP) Channels

The transient receptor potential (TRP) channels are a superfamily of cation channels that act as molecular sensors for a wide range of stimuli, including temperature, mechanical stress, and chemicals from foods and the environment [7,19]. Notably, TRP channels such as TRPA1, TRPV1, and TRPM8 can influence glucose uptake, energy expenditure, and adipose tissue inflammation. Cinnamon-derived compounds are natural agonists for some of these TRP channels, making TRPs key mediators of cinnamon’s metabolic effects [48].

TRPA1 (Transient Receptor Potential Ankyrin 1): TRPA1 is a Ca^2+^-permeable, non-selective cation channel expressed in sensory neurons (especially pain-sensing neurons) and also in some non-neuronal cells in metabolic organs (gut enteroendocrine cells, pancreatic islets, adipose tissue) [7,19]. Cinnamaldehyde is one of the most prominent natural TRPA1 agonists, capable of activating TRPA1 by covalent bonding to the channel’s cysteine-rich N-terminus [19,49]. Activation of TRPA1 initiates a depolarizing influx of cations (Na^+^ and Ca^2+^) in TRPA1-expressing cells, which can lead to various downstream effects.

In the gut, TRPA1 is present in enteroendocrine cells, including ghrelin-producing cells and enterochromaffin cells [7]. Cinnamaldehyde stimulation of TRPA1 in the duodenum has been shown to decrease ghrelin release (ghrelin being an orexigenic hormone) and increase the release of satiety hormones like cholecystokinin (CCK) and GLP-1 [7,13]. In an ex vivo porcine intestine model, cinnamaldehyde triggered significant CCK and GLP-1 secretion via TRPA1 activation [13]. The reduction in ghrelin through TRPA1 is particularly notable, as ghrelin typically stimulates hunger and gastric emptying; TRPA1 activation thus implements an “intestinal brake,” slowing gastric emptying and reducing food intake. In mice, dietary cinnamaldehyde (0.2% of diet) was found to delay gastric emptying and reduce short-term food intake, consistent with these hormonal changes. Collectively, TRPA1 activation in the gut by cinnamon leads to *appetite suppression and improved postprandial glucose tolerance* (due to enhanced GLP-1 and lower ghrelin) [7,50].

In the pancreas, as mentioned in Section 5.1, TRPA1 is expressed in β-cells, and its activation causes membrane depolarization and Ca^2+^ influx, resulting in insulin release [7]. This is an atypical insulin secretory pathway (since it bypasses the need for K-ATP channel closure by glucose metabolism). Cinnamaldehyde’s TRPA1 agonism can thus induce insulin secretion at basal glucose levels, which might help in contexts of impaired first-phase insulin response. Notably, in a study of healthy humans, a single dose of cinnamaldehyde improved glucose tolerance during an oral glucose test without increasing insulin levels excessively, suggesting improved insulin sensitivity rather than simply forcing insulin release, an outcome possibly mediated by TRPA1’s multifaceted actions on gut hormones and β-cells [7,51,52].

In adipose tissue and energy expenditure, TRPA1 is emerging as a metabolic regulator. Activation of TRPA1 in sensory nerves can trigger reflex sympathetic outflow. In one study, chronic cinnamaldehyde feeding did reduce weight gain and improve glucose tolerance, but without a measurable increase in plasma catecholamines [7,53,54]. Instead, cinnamaldehyde was found to *directly* increase the expression of thermogenic and oxidative genes in fat tissue (e.g., CPT1a in white fat, ACSL4 in brown fat), indicating enhanced fatty acid oxidation. TRPA1 activation in adipocytes themselves or in their nerve supply could contribute to this effect. TRPA1 channels are expressed in sensory fibers that innervate adipose tissue, and their stimulation might release CGRP or other neuropeptides that modulate inflammation and metabolism in fat. Also relevant, TRPA1 agonism in brown adipose tissue might not always upregulate UCP1, but still increases energy expenditure through other pathways. In sum, cinnamaldehyde via TRPA1 can promote a shift toward energy expenditure and away from fat storage, aligning with antiobesity effects [7,19].

TRPV1 (Transient Receptor Potential Vanilloid 1): TRPV1 is another well-known TRP channel, famously activated by capsaicin (from chili peppers). TRPV1 is expressed in sensory neurons and many tissues, including skeletal muscle, liver, and adipose. While cinnamon’s cinnamaldehyde is primarily a TRPA1 agonist, some cinnamon components and related compounds can interact with TRPV1. Eugenol, for instance, is structurally a vanilloid and can activate TRPV1 at high concentrations, though it also has partial antagonist or desensitizing effects (it can inhibit capsaicin-induced currents as well) [29,30,55]. The connection between TRPV1 and metabolism has been established largely through capsaicin studies: capsaicin ingestion or chronic activation of TRPV1 is associated with increased energy expenditure, fat oxidation, and reductions in body fat, partly via enhanced sympathetic activity and adipose browning [55]. TRPV1 in sensory nerves of the gut can also slow gastric emptying and increase GLP-1 release, similar to TRPA1, as both channels often coexist in afferent neurons.

Although cinnamon is not as potent on TRPV1 as chili pepper is, there is evidence for TRPV1’s involvement in cinnamon’s effects. A human trial that combined TRPA1 and TRPV1 agonists (cinnamaldehyde and capsaicin) showed additive benefits on fat oxidation. The ingestion of 70 mg cinnamaldehyde and 1 mg capsaicin together significantly increased fat burning compared to placebo, more than either alone in some measures [18,56]. This suggests that TRPV1 activation can complement TRPA1 activation for metabolic improvement. Moreover, one in vitro study found that treating muscle cells with eugenol activated a calcineurin pathway via TRPV1, promoting the formation of oxidative (endurance-type) muscle fibers [57]. This points to a potential role of cinnamon’s eugenol (or maybe cinnamaldehyde cross-activating TRPV1 mildly) in enhancing muscle metabolism and insulin sensitivity.

Thus, while TRPV1 is not the primary target of cinnamon’s main constituent, it likely contributes to the thermal and metabolic sensory effects of cinnamon. Cinnamon ingestion often produces a warming sensation internally; this could be a mild TRPV1 activation in the GI tract or liver. The TRPV1-mediated release of catecholamines and peptides overlaps with TRPA1 pathways, working in tandem to boost metabolic rate and substrate utilization [18,58].

TRPM8 is the cold-sensing channel activated by menthol and cooling agents. Cinnamon does not directly activate TRPM8; in fact, its warming effect is opposite to the cooling of TRPM8. However, TRPM8 agonists have been studied for obesity as well, and in a comparative human trial, a cooling flavor (TRPM8 agonist) was tested alongside cinnamaldehyde and capsaicin. TRPM8 activation did not significantly change energy expenditure or fat oxidation in that acute setting, whereas cinnamaldehyde (TRPA1) did. This underscores that TRPA1 is the dominant TRP channel driving cinnamon’s metabolic effects, rather than TRPM8 [18,59].

TRP channels can influence inflammation. For example, TRPA1 activation on macrophages can induce an anti-inflammatory phenotype via the release of anti-inflammatory neuropeptides, though excessive TRPA1 can also promote neurogenic inflammation in some contexts. In obesity, TRPV1 has been shown to have anti-inflammatory roles; mice lacking TRPV1 developed worse adipose inflammation and insulin resistance, while capsaicin activation of TRPV1 in macrophages can reduce their pro-inflammatory cytokines. Cinnamon’s ability to activate TRPA1 (and possibly TRPV1 to a small degree) in sensory nerves innervating adipose tissue might lead to local release of CGRP or substance P that modulate immune cell function. Interestingly, chronic cinnamaldehyde supplementation in mice attenuated adipose tissue inflammation markers (like reduced macrophage infiltration and inflammatory cytokines) [7,58,60].

In summary, cinnamon phytonutrients engage TRP channels as key mediators (e.g., cinnamaldehyde on TRPA1 (primary) and, to some extent, TRPV1) via eugenol or cross-activation. Through TRPA1 and TRPV1, cinnamon can increase glucose uptake, reduce appetite, enhance thermogenesis, and lower adipose inflammation [7,19]. These channels serve as gatekeepers that convert the chemical stimulus of a spice into hormonal, neural, and metabolic signals, effectively leveraging the body’s sensory system to counter metabolic dysfunction. Cinnamon thereby exemplifies how dietary “nutrient sensing” via TRP channels can be harnessed to influence energy balance and metabolic health.

### 5.3. Ion Channel-Mediated Regulation of Metabolic Homeostasis

The modulation of ion channels by cinnamon compounds translates into several systems-level effects on metabolic homeostasis. By influencing β-cell ion channels (both TRP channels like TRPA1 and classical voltage-gated Ca^2+^ channels), cinnamon can improve the dynamics of insulin release. TRPA1 agonism by cinnamaldehyde has been shown to induce insulin secretion even at baseline glucose, and to upregulate insulin receptor expression in ghrelin-producing cells, suggesting a crosstalk that enhances β-cell responsiveness to nutrients [7,58]. Additionally, there is evidence that chronic cinnamon intake improves β-cell function in vivo. Clinical trials report lowered fasting glucose and HOMA-IR (insulin resistance index) in patients taking cinnamon, sometimes accompanied by a slight increase in insulin levels or improved β-cell sensitivity [61]. By aiding first-phase insulin release (through rapid Ca^2+^ influx via channels) and possibly protecting β-cells from glucotoxicity (through reduced oxidative stress and Ca^2+^ stress), cinnamon helps restore proper insulin signaling needed for homeostasis.

Ion channels in adipocytes (like TRP channels and various K^+^ or Cl^−^ channels) influence adipocyte differentiation, lipolysis, and thermogenesis. Cinnamon’s activation of TRPA1/TRPV1 channels initiates sympathetic signals that enhance lipolysis and fat oxidation [18]. Moreover, as indicated by animal studies, cinnamon can *prevent adipocyte hypertrophy* and promote the browning of white fat. Tamura et al. found that cinnamaldehyde activated brown fat UCP1 and reduced visceral fat in mice. This is an ion channel-mediated outcome: the sensory stimulation by cinnamon engages the β-adrenergic pathway in adipose tissue (likely via TRP channels on sensory nerves that stimulate norepinephrine release) [7,62]. The result is increased energy expenditure by fat cells and reduced storage of lipids. Additionally, improved adipocyte insulin sensitivity has been observed with cinnamon polyphenol treatment [27]. By potentially modulating ion channels that control adipocyte membrane potential and intracellular Ca^2+^ (which is a signal for adipogenesis), cinnamon may tilt the balance away from fat storage and towards fat burning. For example, Ca^2+^-dependent pathways promote adipogenesis; a compound that modulates Ca^2+^ channels could inhibit excessive fat cell formation.

Ion channels not only reside on the plasma membrane but also on organelles. There are mitochondrial ion channels (e.g., calcium uniporters, inner membrane anion channels) that regulate mitochondrial metabolic rates. Enhanced Ca^2+^ influx in response to cinnamon could acutely increase mitochondrial activity (as Ca^2+^ in mitochondria stimulates dehydrogenases in the TCA cycle). In brown adipocytes, increased cytosolic Ca^2+^ via TRP channel activation might secondarily activate pathways leading to UCP1 expression (through Ca^2+^/CAMK signaling). Indeed, one study reported that cinnamaldehyde can induce adipocytes to upregulate genes for fatty acid oxidation and mitochondrial function (CPT1, ACSL) [7,63,64]. This suggests cinnamon’s channel-mediated signaling results in metabolic reprogramming of cells to a higher oxidative state, combating the mitochondrial dysfunction often seen in insulin resistance.

At the whole-body level, the concerted effects of ion channel modulation by cinnamon yield a more favorable energy balance. Reduced appetite (due to gut TRPA1/CCK/GLP-1), increased metabolic rate (due to brown fat activation and muscle substrate use), and improved insulin-driven nutrient uptake all contribute to preventing excess energy storage. In mouse models of diet-induced obesity, cinnamaldehyde supplementation led to lower weight gain without reducing food intake in one study, implying higher energy expenditure [7,54,65]. In humans, acute intake of cinnamaldehyde increased energy expenditure by ~3.6 kcal over 90 min compared to placebo [16]. It also significantly increased fat oxidation rates acutely [18]. These outcomes align well with the idea that ion channels (TRP channels, specifically), when activated, push the body toward burning calories as heat (thermogenesis) and using fat for fuel, a phenotype protective against obesity [19].

Importantly, these ion channel-driven effects do not occur in isolation; they set the stage for synergistic interactions with hormonal signals. For instance, by releasing GLP-1 and insulin, the initial TRPA1 activation by cinnamon ensures that tissues effectively take up and utilize the glucose made available by enhanced oxidation. Conversely, by lowering ghrelin and slowing gastric emptying, it prevents an overshoot of glucose influx that could overwhelm insulin action. Thus, cinnamon’s modulation of ion channels integrates nicely with endocrine control loops to stabilize metabolic homeostasis [7,66].

In summary (Table 1), cinnamon-derived compounds modulate ion channels in multiple organ systems, and these actions converge to improve metabolic health: more insulin when needed, better insulin sensitivity in target tissues, less feeding drive, greater caloric burn, and reduced inflammatory signaling. Ion channels can be considered *upstream “reset buttons”* for the metabolic network, and cinnamon appears to press several of these buttons simultaneously to nudge the system back toward homeostasis.

Ion channels modulated by cinnamon phytonutrients and their roles in metabolic regulation. Cinnamon’s primary effects are mediated by TRPA1 activation (by cinnamaldehyde), with additional contributions from TRPV1 (via eugenol) and general enhancement of Ca^2+^ channel function in secretory cells. The net metabolic outcomes include improved insulin secretion, reduced appetite, increased energy expenditure, and better vascular function, all contributing to antidiabetic and antiobesity effects [7,18].

## 6. GPCR-Targeted Metabolic Actions of Cinnamon Phytonutrients

**Figure 4:**
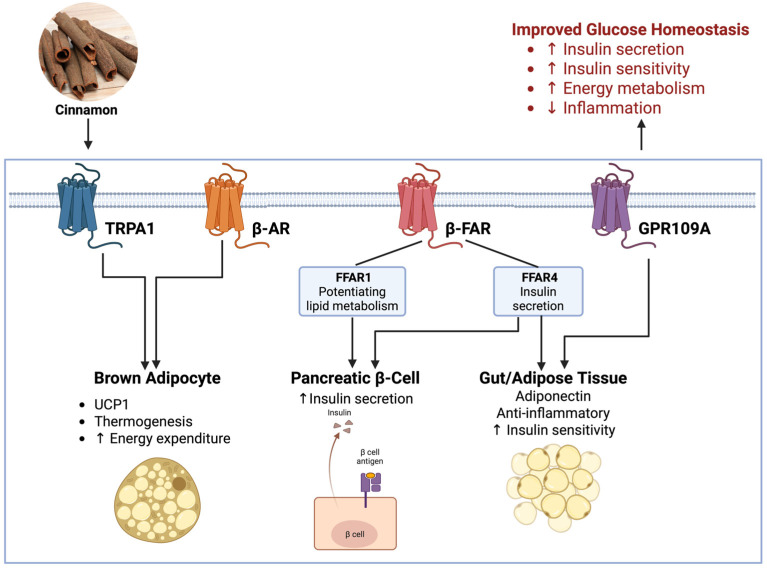
GPCR-mediated metabolic pathways influenced by cinnamon phytonutrients. Cinnamon modulates metabolic GPCR signaling indirectly by stimulating endogenous ligands such as GLP-1, catecholamines, and cinnamic acid, thereby enhancing insulin secretion, lipid oxidation, thermogenesis, and anti-inflammatory responses. Figure created via Biorender Premium License by Fahrul Nurkolis. ↑ Increase; ↓ decrease.

GPCRs are another major class of membrane signaling proteins through which cinnamon-derived nutrients can exert metabolic effects (Figure 4). Unlike ion channels that directly mediate ionic flux, GPCRs activate intracellular signaling cascades (via G proteins and β-arrestins) to modulate cellular responses. Many metabolites and nutrients (fatty acids, amino acids, bile acids, etc.) have their own GPCRs, often termed “metabolite-sensing receptors” [1]. In metabolic disease therapy, a few GPCRs have gained prominence (e.g., the GLP-1 receptor targeted by incretin mimetics, or β-adrenergic receptors targeted for obesity in experimental drugs), but numerous others remain underutilized. Cinnamon phytonutrients may influence some of these GPCR pathways either by acting as ligands (in native or metabolized form) or by modulating GPCR signaling bias and sensitivity [10,67].

### 6.1. GPCRs in Glucose and Lipid Metabolism

Several GPCR families play central roles in maintaining glucose and lipid homeostasis [1]. First, adrenergic receptors (β-ARs) are activated by catecholamines (epinephrine and norepinephrine) and are critical in metabolic regulation. β-AR activation in adipose tissue stimulates lipolysis (breakdown of triglycerides) and thermogenesis (especially β_3 in brown fat promotes UCP1-mediated heat production) [1]. Cinnamon, indirectly, can influence β-adrenergic pathways. As discussed, TRPA1 activation by cinnamaldehyde may provoke sympathetic nerve firing and release of norepinephrine in adipose tissue. This would in turn activate β-ARs on adipocytes, enhancing lipolysis and energy expenditure. Indeed, one mouse study reported that cinnamaldehyde’s antiobesity effect could be associated with β-adrenergic activation: Tamura et al. found increased brown fat UCP1 and reduced fat mass in cinnamaldehyde-treated mice, effects typically downstream of β_3-AR stimulation [7,18,53]. Another study noted improved plasma lipid profiles and reduced visceral fat with cinnamon, which could result from increased β-adrenergic-driven fat utilization. Clinically, cinnamon supplementation in humans has led to decreased triglycerides and cholesterol, consistent with enhanced lipid catabolism [6]. While cinnamon’s compounds do not bind β-ARs directly (they are not similar to catecholamines structurally), by triggering catecholamine release via sensory mechanisms, they indirectly engage β-AR signaling. This suggests cinnamon could complement or enhance the effect of exercise or cold exposure (natural activators of β-adrenergic pathways) as a nutraceutical stimulant of energy expenditure.

Free Fatty Acid Receptors (FFARs) are GPCRs that sense fatty acids; notable ones in metabolism include GPR40 (FFAR1) and GPR120 (FFAR4). GPR40 is expressed on pancreatic β-cells and incretin-secreting cells; it is activated by medium-to-long chain fatty acids and potentiates glucose-stimulated insulin secretion [1]. GPR120 is found on macrophages and adipose tissue, responding to unsaturated fatty acids (especially omega-3s) and mediating anti-inflammatory and insulin-sensitizing effects [20]. While cinnamon compounds are not fatty acids, cinnamon intake could influence these GPCRs indirectly. One possibility is that cinnamon alters lipid metabolism or gut microbiota such that more endogenous ligands for these receptors are produced. For example, cinnamon has been shown to increase circulating levels of certain free fatty acids or improve their tissue uptake, which might modify GPR40 signaling on β-cells [6]. Enhanced GPR40 activity would improve insulin secretion in response to meals. On the GPR120 side, any reduction in inflammation by cinnamon could be through pathways converging on GPR120 activation. A relevant clue: cinnamaldehyde’s metabolite cinnamic acid can bind to GPR109A (discussed below), which triggers a cascade somewhat analogous to GPR120’s (increasing adiponectin, anti-inflammatory). Cinnamon’s metabolic actions resonate with those GPCRs’ known effects, such as improved insulin output (GPR40-like) and reduced adipose inflammation (GPR120-like) [1,20].

GLP-1 Receptor (GLP-1R), located on pancreatic β-cells and brain neurons, binds the incretin hormone GLP-1 and powerfully enhances insulin secretion while suppressing glucagon, thereby lowering blood glucose [1]. Cinnamon has been observed in clinical studies to raise GLP-1 levels postprandially [68,69]. Mechanistically, as noted earlier, cinnamaldehyde via TRPA1 provokes GLP-1 release from intestinal L-cells [13]. The increased endogenous GLP-1 can then act on GLP-1Rs to amplify insulin release and possibly to slow gastric emptying and reduce appetite (central GLP-1 effects). In a small trial, people consuming cinnamon showed higher GLP-1 and lower insulin spikes, suggesting improved insulin efficiency [68,70]. By harnessing the incretin pathway, cinnamon indirectly activates GPCR signaling in β-cells (GLP-1R), which could explain improved glycemic control. This highlights a theme: cinnamon compounds often work by modulating endogenous GPCR ligands (hormones/neurotransmitters) rather than binding GPCRs in a lock-and-key fashion themselves.

Numerous GPCRs detect metabolic byproducts, e.g., GPR109A for niacin (and ketone body β-hydroxybutyrate), GPR91 for succinate, GPR41/43 for short-chain fatty acids, and TGR5 for bile acids. GPR109A is expressed in adipose tissue and immune cells; its activation by niacin causes a flushing response and an increase in adiponectin with simultaneous inhibition of lipolysis. Interestingly, cinnamic acid (the oxidized metabolite of cinnamaldehyde) is an agonist of GPR109A. A study by Kopp et al. (2014) [71] showed that cinnamic acid activated GPR109A in adipocytes, leading to elevated adiponectin secretion and AMPK activation. This is significant because adiponectin is an insulin-sensitizing, anti-inflammatory adipokine often reduced in obesity. By engaging GPR109A, cinnamon might improve adiponectin levels and thereby enhance systemic insulin sensitivity and fatty acid oxidation. In fact, the same SciRep 2015 study postulated that some long-term benefits of cinnamaldehyde were due to its conversion to cinnamic acid and subsequent GPR109A activation [7,71]. Other GPCRs such as TGR5 (a bile acid receptor that can increase energy expenditure via thyroid hormone activation and GLP-1 release) might also be influenced by cinnamon through microbiome interactions that alter bile acid pools, though direct evidence is not yet available [72,73].

In summary, cinnamon’s impact on GPCRs in metabolism is both indirect and multi-modal: it triggers the release of or conversion to endogenous agonists (GLP-1, catecholamines, adipokines) that then act on GPCRs like GLP-1R, β-ARs, and GPR109A; and it potentially biases signaling outcomes (for instance, favoring anti-inflammatory GPCR pathways over pro-inflammatory ones). The result is an array of GPCR-mediated benefits: higher postprandial insulin through GLP-1R, greater fat breakdown and thermogenesis via β-ARs, and improved adipose hormonal profile via GPR109A and possibly GPR120-like mechanisms [1,7]. This orchestrated tuning of GPCR signals underlies much of cinnamon’s nutraceutical value in metabolic disease.

### 6.2. Biased GPCR Signaling in Metabolic Control

Biased signaling is the phenomenon where different ligands or modulators of the same GPCR preferentially activate certain intracellular pathways (G protein vs. β-arrestin, or even specific G protein subtypes) [1,74,75]. Biased agonism allows fine-tuning of receptor outcomes, which can be leveraged to maximize therapeutic effects while minimizing side effects. In metabolic regulation, biased GPCR signaling is highly relevant. For example, β-arrestin-biased agonists at the β_3-adrenergic receptor could induce beneficial thermogenesis without excessive cardiovascular stimulation; biased GLP-1R agonists might promote weight loss pathways more than those causing nausea.

Nutraceuticals may act as biased modulators of GPCRs. They often are partial agonists or allosteric modulators rather than full agonists, which inherently gives them a bias profile often favoring one signaling arm. Many plant polyphenols and metabolites act as partial agonists at GPCRs. For instance, the GPR40 agonist fasiglifam (TAK-875), though synthetic, was a partial agonist that preferentially triggered G_q-Ca^2+^ signaling with little β-arrestin recruitment [1]. If a cinnamon-derived ligand (like cinnamic acid at GPR109A) is a partial agonist, it might similarly favor one pathway. In the case of GPR109A, β-hydroxybutyrate (an endogenous ligand) is biased toward anti-lipolytic G_i signaling, whereas niacin strongly recruits β-arrestin, causing the flush. Cinnamic acid’s profile is not fully known, but its beneficial effects (increasing adiponectin via AMPK) suggest it might avoid strong β-arrestin recruitment that leads to prostaglandin release (the cause of flushing) [7,71]. Thus, cinnamic acid could be a biased GPR109A agonist that achieves metabolic benefits (AMPK activation, adiponectin up) without the side effect profile of niacin, an intriguing possibility for therapy.

Polyphenols often bind GPCRs at allosteric sites, tweaking the receptor’s response to its primary ligand. This can imbue bias; for example, a polyphenol might enhance G protein signaling of a receptor while blocking β-arrestin binding. If cinnamon polyphenols bind to, say, the β_2-adrenergic receptor allosterically, they might promote one pathway. Although speculative, one could envision a scenario where cinnamon extract ingestion leads to biased β-adrenergic effects, e.g., promoting skeletal muscle glucose uptake (perhaps through β-arrestin signaling, which has been implicated in insulin sensitivity in some contexts) without excessive heart rate increase (which is mostly G_s-cAMP mediated). Supporting this concept, β-arrestin-biased ligands for β-adrenergic receptors (like carvedilol at β_1-AR) can have unique beneficial effects [1,76,77].

GLP-1 receptor can signal via multiple G proteins and β-arrestin; different GLP-1 analogs have varying degrees of bias, which might affect their tendency to cause tachycardia or nausea. Nutrients that stimulate native GLP-1 release (like cinnamaldehyde) will engage the natural hormone, which has its own signaling profile. However, if cinnamon also influences other gut receptors (like taste receptors on enteroendocrine cells), it could bias the mix of incretins released (GLP-1 vs. GIP). This “bias” in hormone release could be due to cinnamon activating TRPA1 on L-cells (GLP-1 producers) but not significantly on K-cells (GIP producers), thereby skewing the incretin response toward GLP-1 [78,79].

GPCRs like GPR120 on macrophages can signal through G_q or β-arrestin pathways, where β-arrestin-2 is crucial for anti-inflammatory effects. A biased agonist that favors β-arrestin (as some resolvins do for GPR32, for example) has enhanced anti-inflammatory benefit. If a cinnamon metabolite or constituent can selectively promote the β-arrestin arm of GPR120 or related receptors, it would yield greater insulin sensitization (through macrophage M2 polarization and adipose tissue health) [20]. We know cinnamon reduces markers of inflammation and oxidative stress in metabolic syndrome patients; perhaps this involves biased activation of GPCRs that control inflammation (like more IL-10 release vs. TNF-α from macrophages) [6,80].

In conclusion, while explicit examples of biased GPCR signaling by cinnamon phytonutrients are still being investigated, the concept is important. We can already appreciate that cinnamon’s effects are not “all-or-nothing” stimulations of GPCRs but rather nuanced modulations. This nuance is likely the reason cinnamon can, for instance, lower glucose and improve lipids without causing hypoglycemia or drastic BP changes. It modulates GPCR pathways within physiological bounds. Going forward, identifying how cinnamon compounds bias key receptors (GLP-1R, β-ARs, GPR109A, GPR120, etc.) will help optimize the use of cinnamon or its analogues in metabolic therapies. Biased signaling presents an opportunity to fine-tune metabolism by engaging receptors in an optimal way that maximizes therapeutic benefits (insulin secretion, fat oxidation, anti-inflammation) while minimizing side effects (excessive adrenergic stimulation, flushing, GI upset).

### 6.3. Anti-Inflammatory and Insulin-Sensitizing Effects

A hallmark of metabolic diseases is chronic inflammation in tissues like visceral fat, liver, and the pancreas. This metaflammation impairs insulin signaling and perpetuates metabolic dysfunction. Both ion channels and GPCRs on immune cells and metabolic cells contribute to inflammatory pathways. Cinnamon’s phytonutrients exhibit notable anti-inflammatory properties, which, in the context of metabolic disease, translate to improved insulin sensitivity [12,81,82].

One major site of metabolic inflammation is the adipose tissue, where interactions between adipocytes and infiltrating macrophages create a vicious cycle of cytokine production (e.g., TNF-α, IL-6) that induces insulin resistance. GPCRs on these cells (such as GPR120 on macrophages and ADRB2 on adipocytes) are crucial modulators of inflammation. Activation of GPR120 by omega-3 fatty acids, for example, triggers a switch in macrophages to an anti-inflammatory state and enhances insulin sensitivity in adipocytes [1,20,83]. Cinnamon effects mirror some outcomes of GPR120 activation, namely, suppression of pro-inflammatory cytokines and improved insulin action. In obese rodent models, cinnamon supplementation reduced expression of TNF-α and MCP-1 in adipose tissue, indicating fewer M1-type (pro-inflammatory) macrophages and a more M2-like, anti-inflammatory environment [7,84]. This could be mediated by GPCR pathways: possibly cinnamon’s enhancement of adiponectin (via GPR109A as discussed) plays a role, since adiponectin has anti-inflammatory effects on macrophages. Another angle is macrophage-adipocyte crosstalk through sensory nerves.

TRPV1 on adipose-resident sensory neurons has been implicated in curbing adipose inflammation; capsaicin-mediated TRPV1 activation can lead to the release of CGRP, which suppresses macrophage inflammatory cytokine production. If cinnamaldehyde activates those same sensory fibers via TRPA1/TRPV1, it can induce a neuroimmune reflex dampening adipose inflammation. Indeed, TRPA1 activation can cause release of anti-inflammatory peptides in some contexts [7,58]. The result is improved insulin signaling in adipocytes because the inflammatory blockade on insulin receptors is lifted.

Systemically, cinnamon intake in humans has been associated with reduced markers of inflammation, such as C-reactive protein (CRP). The anti-inflammatory effect can also be partially attributed to the direct antioxidant action of polyphenols (reducing NF-κB activation), but GPCR signaling is a key upstream regulator of inflammation that cinnamon influences. For example, the NF-κB pathway in macrophages can be inhibited by PKA (downstream of G_s-coupled receptors like β-AR); thus, if cinnamon causes a mild β-adrenergic activation in immune cells, it could tilt them to a less inflammatory phenotype. Also, some cinnamon flavonoids may bind to estrogen-related GPCRs or PPARγ (though PPARγ is not a GPCR, its activation in macrophages also reduces inflammation) [6,85,86].

Insulin-sensitizing effects of cinnamon have been noted in many studies, sometimes without large changes in insulin levels, suggesting improved insulin action at the tissue level [61]. One mechanism is increased adiponectin, which we have linked to GPR109A activation by cinnamic acid [7]. Adiponectin acts on its receptors (which have some GPCR-like signaling aspects) to activate AMPK in muscle and liver, enhancing insulin sensitivity and fatty acid oxidation. In a clinical trial, cinnamon supplementation raised adiponectin concentrations in patients with metabolic syndrome [73,87]. This hormone likely contributes significantly to cinnamon’s insulin-sensitizing outcomes by promoting skeletal muscle glucose uptake and suppressing hepatic gluconeogenesis (via AMPK).

There is also an interplay between GPCR and insulin receptor signaling. Chronic inflammation can lead to serine phosphorylation of insulin receptor substrate (IRS) proteins, blunting insulin signals. GPCRs like TNF receptors are not GPCRs (they are cytokine receptors) but GPCRs such as GPR120, when activated, can inhibit NF-κB and JNK pathways that cause those serine phosphorylations [20]. Therefore, cinnamon’s ability to activate anti-inflammatory GPCR pathways (like GPR109A, possibly GPR120 indirectly) helps preserve insulin signaling integrity.

Furthermore, liver inflammation (NASH) involves GPCR crosstalk, e.g., GPCR91 (succinate receptor) on stellate cells triggers fibrosis in response to signals from dying hepatocytes [14]. By improving overall metabolic health and reducing adipose lipolysis (excess FFA delivery to the liver), cinnamon likely indirectly reduces these GPCR-mediated fibrogenic signals too.

In summary, cinnamon’s anti-inflammatory and insulin-sensitizing effects stem from a combination of its direct antioxidant/anti-inflammatory actions and its modulation of membrane receptors that govern inflammation. It suppresses “metaflammation” by: (1) activating GPCR pathways that yield anti-inflammatory mediators (e.g., adiponectin via GPR109A; ω3-like signaling via GPR120 analogues); (2) triggering neuropeptide release via TRP channels that reduce local inflammation in adipose tissue; and (3) improving the balance of pro- vs. anti-inflammatory immune cells in metabolic tissues. The result is enhanced insulin sensitivity: muscle and liver respond better to insulin, and pancreatic β-cells are under less inflammatory stress and thus function better [7,20].

## 7. Ion Channel–GPCR Crosstalk in Metabolic Tissues

**Figure 5:**
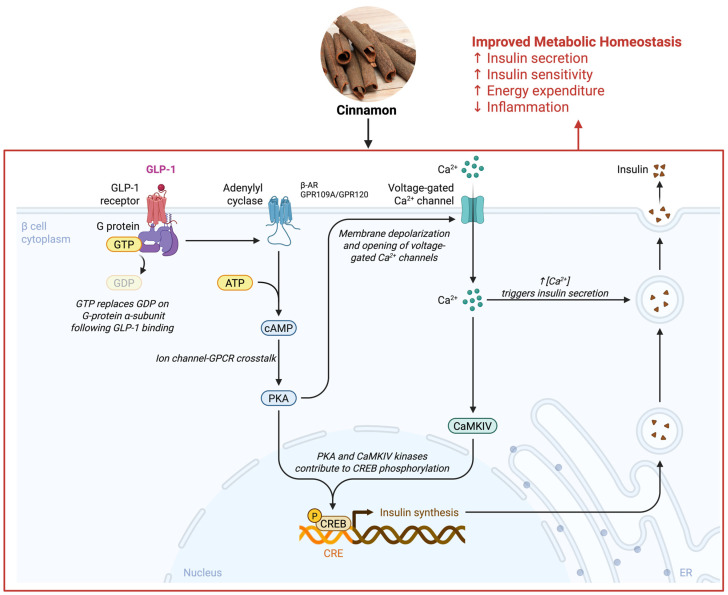
Crosstalk between ion channels and GPCRs in metabolic regulation. Cinnamon-derived phytonutrients orchestrate metabolic homeostasis through coordinated ion channel–GPCR signaling networks across gut, pancreas, adipose tissue, and nervous system, illustrating a systems-level mechanism underlying their pleiotropic metabolic benefits. Figure created via Biorender Premium License by Fahrul Nurkolis. ↑ Increase; ↓ decrease.

Ion channels and GPCRs do not operate in isolation; they engage in extensive crosstalk within cells and across organ systems. This crosstalk is particularly evident in metabolic tissues like pancreatic islets, adipose tissue, liver, and the nervous system controlling metabolism (Figure 5).

One classic example of ion channel–GPCR interplay is in the pancreatic β-cell: the process of insulin secretion involves both ion channel activity and GPCR modulation. When glucose triggers β-cell depolarization through K-ATP channel closure and L-type Ca^2+^ channel opening (ion channels), the resulting Ca^2+^ influx not only directly causes exocytosis but also can be amplified by GPCR signaling. GPCR-induced Ca^2+^ flux in β-cells occurs via receptors like the GLP-1R (a G_s-coupled receptor). Activation of GLP-1R by incretins raises cAMP, which, via PKA and EPAC2, enhances the opening of voltage-gated Ca^2+^ channels and mobilizes insulin granules [1,88]. Cinnamon’s ability to increase GLP-1 release ties directly into this cAMP–Ca^2+^ synergy: more GLP-1 leads to heightened cAMP in β-cells, which potentiates Ca^2+^ channel activity and exocytotic machinery, thereby boosting insulin secretion on top of the direct TRPA1-mediated Ca^2+^ influx [7,89]. Cinnamon (via TRPA1) provides an initial Ca^2+^ signal and, via GPCR (GLP-1R), strengthens that signal as a one-two punch for insulin release.

Adipocytes express GPCRs (like β_adrenergic receptors, cannabinoid receptors, adiponectin receptors) that regulate lipolysis and glucose uptake. Ion channels on adipocytes (like TRPV4, Ca^2+^ channels, K^+^ channels) can modulate membrane potential and intracellular Ca^2+^, influencing differentiation and metabolism. For example, β_3-adrenergic GPCR activation in brown adipocytes leads to cAMP production, which not only triggers lipolysis but also can directly modulate certain Ca^2+^-activated ion channels or transcription factors. Calcium-dependent kinases may be involved in the gene expression cascade for browning. TRP channels in adipocytes (like TRPV2 and TRPV4) respond to cellular stress and can activate MAPK pathways. TRPV4, when activated by a fatty acid metabolite, can initiate an inflammatory gene program; but if a GPCR (like GPR120) simultaneously signals for anti-inflammatory actions (via β-arrestin), it can counteract that. Thus, adipocyte function is a balance of GPCR and ion channel inputs, which cinnamon helps tip in a positive direction: TRPA1 activation in sensory nerves reduces local adipose inflammation (affecting macrophage GPCRs for cytokines), and concurrently, GPCR109A activation by cinnamic acid in adipocytes triggers AMPK, improving mitochondrial function. This kind of network ensures that adipocytes do not just indiscriminately release fatty acids (which cause insulin resistance), but rather increase oxidation of fats (due to AMPK and UCP1 upregulation) [7,90,91].

Feedback loops are common in these networks. For instance, if cinnamon causes a surge in GLP-1 (GPCR ligand), insulin release will go up, lowering blood glucose. Lower glucose will then cause β-cells to reduce electrical activity (ion channels hyperpolarize as ATP falls back to baseline), preventing excessive insulin and avoiding hypoglycemia. Similarly, activation of TRPA1 in the gut (ion channel) slows gastric emptying, which then modifies the nutrient stimulus for GPCRs in the gut and pancreas (like GPR40 sensing fatty acids, or sweet taste receptors on K/L cells). The system has built-in brakes and throttles: ion channels often provide rapid, acute signals, whereas GPCR pathways can have more sustained modulatory effects. Cinnamon’s multi-target action engages both, but the body’s feedback ensures synergy rather than overload [42,43,61].

From a systems biology perspective, one can view metabolism as a complex circuit where membrane proteins are the control knobs. Ion channels are like fast switches, and GPCRs are like dimmer knobs adjusting signal intensity. Cinnamon, with its suite of compounds, touches multiple control knobs at once, a form of network pharmacology. For example, a slight increase in β-cell Ca^2+^, a modest increase in cAMP plus a reduction in inflammatory tone can together revive insulin output dramatically. Systems analyses (including network pharmacology models) have begun to confirm that cinnamon’s components target diverse protein networks rather than one receptor or enzyme [8,92,93].

Ion channel–GPCR crosstalk also underlies neuro-metabolic integration. The autonomic nervous system uses GPCRs (like adrenergic, muscarinic receptors) to control organ function and ion channels to fire nerve impulses. Cinnamon’s sensory activation (TRP channels on vagal and splanchnic nerves) sends afferent signals to the brainstem, which then, via efferent autonomic fibers, adjusts insulin secretion, gut motility, and thermogenesis, all mediated by GPCRs on target organs (e.g., adrenergic receptors on brown fat, muscarinic on islets). Such cross-communication between sensory ion channels and efferent GPCR signals illustrates the complexity of cinnamon’s impact. It does not act only locally; it recruits the neuroendocrine axis. For instance, TRPA1 activation in the gut can ultimately lead to sympathetic output that hits β-AR in fat, a full circuit from ion channel to GPCR across different organs [7,94].

In conclusion, the crosstalk between ion channels and GPCRs in metabolic tissues is a central theme in understanding integrative physiology. Cinnamon’s multifaceted influence on this network is truly an outstanding aspect; it underscores how a single natural agent can harmonize multiple signals: electrical, chemical, neural, and hormonal. This systems-level approach is what likely allows cinnamon to have tangible benefits in complex disorders like metabolic syndrome, where hitting one target often is not enough. By modulating ion channel–GPCR circuits, cinnamon helps rewire dysfunctional metabolic networks back towards a healthier state.

## 8. Translational Potential in Metabolic Diseases

**Figure 6:**
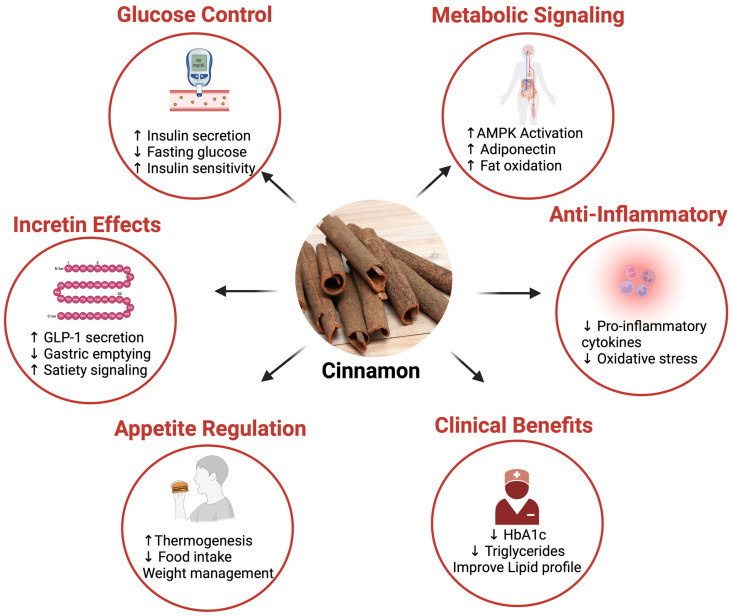
Translational and precision nutrition perspectives of cinnamon-based membrane signaling modulation. By targeting ion channel–GPCR networks, cinnamon phytonutrients may complement existing antidiabetic therapies and support personalized metabolic interventions guided by omics-based precision nutrition strategies. Figure created via Biorender Premium License by Fahrul Nurkolis. ↑ Increase; ↓ decrease.

The insights into how cinnamon phytonutrients modulate ion channels and GPCRs suggest several translational opportunities (Figure 6). Cinnamon, as a nutraceutical or adjunct therapy, could be strategically combined with existing treatments for metabolic diseases to enhance outcomes. Moreover, practical considerations around dosing, formulation, and safety will determine how best to implement cinnamon’s benefits in clinical settings. In this section, we discuss the synergy of cinnamon with conventional antidiabetic therapies and address issues of dosage, standardization, and safety that are crucial for its use in medicine.

### 8.1. Combination with Antidiabetic Therapies

Given cinnamon’s mechanisms (insulin secretion enhancement, insulin sensitivity improvement, and appetite regulation), it makes sense to combine it with standard antidiabetic drugs, which often work through different pathways. Metformin primarily acts in the liver to reduce gluconeogenesis and improve insulin sensitivity (partly via AMPK activation). It also has systemic effects like altering gut microbiota and incretin levels. Cinnamon overlaps in some areas (AMPK via adiponectin, incretins via GLP-1) but also adds unique actions (e.g., TRPA1-mediated insulin release). In a clinical study on women with polycystic ovary syndrome (PCOS, often accompanied by insulin resistance), cinnamon (1.5 g/day) and metformin (1500 mg/day) were tested against each other and a placebo. Both cinnamon and metformin significantly decreased insulin resistance (HOMA-IR) compared to placebo, and the reduction in HOMA-IR with cinnamon was similar to that with metformin [61]. This suggests that adding cinnamon to metformin might further reduce insulin resistance. Indeed, that trial hinted at possibly additive effects: both together might achieve more than either alone, although it was not a combination arm per se. However, other studies in type 2 diabetes patients have explicitly combined cinnamon with metformin. Some reports indicate improved HbA1c and fasting glucose with the addition of cinnamon to metformin therapy, as well as better lipid profiles [95,96]. Mechanistically, while metformin activates AMPK mainly in the liver, cinnamon can activate AMPK in adipose via GPR109A-adiponectin and in muscle via improved Ca^2+^ signaling [7].

GLP-1 analogs (like liraglutide, semaglutide) are highly effective but can cause GI side effects at higher doses. Cinnamon’s ability to elevate endogenous GLP-1 might allow a lower dose of injected GLP-1 RA or enhance its effect. Moreover, cinnamon also affects other pathways beyond GLP-1 (like insulin sensitivity, which GLP-1 RAs less directly address). There is no direct clinical trial of cinnamon with GLP-1 RAs yet, but one could envision that pairing them might improve glycemic control and weight loss more than either alone. Similarly, DPP-4 inhibitors (which prevent GLP-1 breakdown) could see an amplified effect if more GLP-1 is secreted due to cinnamon. Essentially, cinnamon can be thought of as a secretagogue for incretins working upstream, whereas DPP-4i works downstream. The two could synergize to raise incretin levels significantly, which would then strongly stimulate insulin and satiety [12,97].

For patients on insulin therapy, cinnamon might help by reducing the required insulin dose through its insulin-sparing effect. Improved insulin sensitivity and some endogenous insulin release from cinnamon could lower exogenous insulin needs and possibly stabilize blood sugar between doses. With sulfonylureas (which force insulin out via K-ATP channel closure), cinnamon’s TRPA1 route offers an alternative way to augment insulin that might be glucose-dependent (since TRPA1 in β-cells likely needs some basal metabolic activity). Notably, glibenclamide, being a TRPA1 agonist as well, means sulfonylurea and cinnamon might overlap in stimulating TRPA1. Interestingly, this could either be additive or, if saturating the same mechanism, just redundant. But since cinnamon also increases incretins and adiponectin, it would still add beyond the sulfonylurea’s pure insulin secretion action [19].

SGLT2 inhibitors cause glycosuria to lower glucose. They also modestly induce ketogenesis and weight loss. Cinnamon could complement by further curbing postprandial spikes (via slower gastric emptying and more insulin) and by addressing insulin resistance (which SGLT2i does not directly do). There is also potential benefit in renal health (cinnamon’s antioxidant effects might protect kidneys, which is relevant as SGLT2i puts more load on kidneys to excrete glucose). Though data is lacking, this combination might be beneficial for comprehensive management [98,99]. Exercise activates many of the same pathways as cinnamon (AMPK, TRP channels via muscle heating, adrenaline release). Combining cinnamon supplementation with exercise could theoretically amplify exercise’s benefits. For example, exercise increases GLP-1 a bit and muscle insulin sensitivity; cinnamon could add to that, potentially improving endurance as one study on eugenol indicated via TRPV1 in muscle. From a patient perspective, using cinnamon as a nutraceutical could be an easy add-on to lifestyle changes, reinforcing them through subtle physiological effects [21,32].

Overall, combination approaches are promising because metabolic diseases typically require multifactorial management. Cinnamon’s broad mechanism profile means it can fill gaps in current therapy. It addresses postprandial glucose (like acarbose would), insulin secretion (like sulfonylureas but more gently), insulin sensitivity (like metformin or TZDs without their side effects, one hopes), and appetite/weight (like GLP-1 RAs, though milder). Thus, adding cinnamon might allow dose reduction of pharmaceutical agents (minimizing side effects) or achievement of targets that single drugs cannot reach. A review of multiple trials concluded that cinnamon as an adjunct improved glycemic and lipid parameters without significant adverse effects, underscoring its potential in integrative treatment plans [100,101].

### 8.2. Nutritional Dosing and Safety

Translating the benefits of cinnamon to clinical practice requires careful attention to dosing, standardization, and safety. While cinnamon is natural, it is not without potential risks (particularly due to coumarin content). Clinical studies have used a range of doses, commonly 1–6 g of cinnamon powder per day, often around 3–4 g/day in divided doses, to achieve metabolic benefits [70,100]. Meta-analyses suggest that doses in this range can lead to modest but significant reductions in fasting glucose (~10–15 mg/dL), HbA1c (~0.5%), and improvements in cholesterol and triglycerides [100]. For perspective, 1 teaspoon of ground cinnamon is roughly 2.5–3 g. It is noteworthy that Ceylon cinnamon vs. Cassia cinnamon may have different potencies; much of the research used Cassia cinnamon (higher coumarin content). Ceylon (true) cinnamon has less coumarin and is considered safer for long-term use at higher doses, although its cinnamaldehyde content might be slightly lower. Standardized extracts (like those providing a specific amount of polyphenols or cinnamaldehyde) have been tested as well, sometimes in capsule form (e.g., 500 mg capsules standardized to cinnamaldehyde content, taken thrice daily) [61]. These appear effective and may be preferable for consistent dosing.

For medicinal use, standardization is key either by specifying the type (*C. cassia* vs. *C. verum*) or by extracting and quantifying active constituents (like >60% cinnamaldehyde in essential oil, or a certain percentage of polyphenols in aqueous extract). Nutraceutical products often list cinnamon extract with a certain extract ratio (e.g., 10:1) and sometimes the amount of polyphenol content. Cinnulin PF is one patented water-soluble cinnamon extract that is coumarin-free and used in studies at ~250 mg twice daily; it has shown some efficacy in improving fasting glucose and body composition in pre-diabetics. Using such extracts can mitigate safety issues related to coumarin, since coumarin is mostly in the fat-soluble fraction (essential oil) that may not be present in water extracts [27]. On the other hand, cinnamaldehyde is oil-soluble, so water extracts might contain less of it; thus, a combination or broad-spectrum extract might be ideal to capture both water-soluble polyphenols and lipophilic aldehydes, while removing just the unwanted coumarin.

The biggest safety concern with chronic high-dose cinnamon (especially Cassia) is coumarin-induced hepatotoxicity. From a translational perspective, safety considerations are critical when positioning cinnamon as a nutraceutical. Coumarin can cause liver enzyme elevations and even liver injury in sensitive individuals if taken in excess. The tolerable daily intake (TDI) for coumarin is about 0.1 mg per kg body weight, which for a 70 kg person is ~7 mg coumarin per day. Cassia cinnamon powder can contain about 2–5 mg of coumarin per gram (though it varies widely). This means that taking 3–4 g of Cassia cinnamon could provide near or above the TDI of coumarin on a chronic basis. Ceylon cinnamon, by contrast, has very low coumarin (trace amounts), making it much safer for high-dose usage. Therefore, if patients intend to take cinnamon daily for months or years, Ceylon cinnamon or a coumarin-free extract is strongly recommended to avoid liver issues [27]. Patients with pre-existing liver disease should be particularly cautious; some case reports note hepatotoxicity in such individuals consuming cinnamon supplements. Monitoring liver enzymes might be prudent if using high-dose cinnamon capsules long-term.

Cinnamon is generally well tolerated. GI discomfort is possible in some (large amounts can be a gastric irritant, though interestingly, it slows gastric emptying, which might cause feelings of fullness/bloating initially). Allergic reactions are rare but can occur (cinnamon is a common spice allergen for some, and cinnamal is a known contact allergen in cosmetics). Because cinnamon lowers blood sugar, there is a theoretical risk of hypoglycemia if combined with diabetes medications; in practice, since its effect is moderate, this is not commonly reported, but healthcare providers should be aware and perhaps adjust diabetes medication dosages as needed when a patient starts cinnamon to avoid too-low sugars [61]. On the positive side, cinnamon’s effect is glucose-dependent (working more when glucose is high, via incretins and β-cell stimulation), so hypoglycemia risk remains low [7,61].

Cinnamaldehyde is rapidly metabolized (largely to cinnamic acid, then further to benzoate and hippurate, which are excreted in urine). Polyphenols from cinnamon have limited absorption in the small intestine but are metabolized by colonic bacteria to smaller phenolics (some of which may be active). The timing of cinnamon intake relative to meals might influence its efficacy. Taking it with or just before meals seems logical to maximize the impact on postprandial glucose (through delaying gastric emptying and enhancing insulin secretion). Indeed, studies that gave cinnamon prior to a glucose challenge saw better glucose handling [7,39,40]. For supplements, dividing the dose with meals (e.g., 1 g each with breakfast, lunch, dinner) could produce a steadier effect. The gut microbiome plays a role in transforming cinnamon compounds; interestingly, byproducts like phenylpropionate could have their own GPCR targets in the gut. Some research in animals indicates cinnamon can beneficially alter the microbiome composition (increasing beneficial bacteria), which might indirectly improve metabolism [102,103].

Reaching therapeutic doses might be challenging with diet alone because 3–4 g is a lot of cinnamon to incorporate daily just via cooking. Cinnamon tea (steeping a cinnamon stick) is one approach some use; it provides polyphenols with minimal coumarin (since coumarin is not very water-soluble). A cup of strong cinnamon tea before meals could be a gentle way to get some effect. For a more consistent approach, standardized capsules are likely better. Importantly, patients should be educated that more is not always better; megadosing above the studied range could raise risk without a clear added benefit [82,104,105].

In conclusion, nutritional use of cinnamon for metabolic benefits is feasible and safe when done properly. Selecting a low-coumarin source (Ceylon cinnamon or aqueous extract) and using doses in the 1–6 g/day range (or equivalent extract) can harness its benefits while minimizing risk. It is economical and accessible a significant advantage for a worldwide problem like diabetes. Nonetheless, patients and practitioners should treat it with the same respect as any adjunct therapy: monitor its effects, watch for rare side effects, and ensure it complements the overall treatment plan. With these safeguards, cinnamon can be a valuable part of the metabolic toolkit, bridging lifestyle and pharmacotherapy.

## 9. Future Directions

Research into cinnamon’s role in metabolic health is part of a broader exploration of food as medicine. The complex interaction of cinnamon phytonutrients with multiple targets invites modern scientific approaches to fully elucidate and harness these effects. Future directions include leveraging cutting-edge omics technologies to understand cinnamon’s molecular impact, and pursuing personalized nutrition strategies to maximize its benefits for individuals. Here, we outline some promising avenues:

### 9.1. Omics-Guided Precision Nutrition

Advances in genomics, proteomics, metabolomics, and related fields (collectively, *omics*) offer new ways to dissect how cinnamon works and for whom it works best. By profiling metabolites in blood or urine, researchers can map out the biochemical changes after cinnamon intake. For instance, metabolomic studies have found that cinnamon supplementation leads to changes in pathways like fatty acid β-oxidation and amino acid metabolism [73,103]. Identifying specific metabolite signatures (like an increase in hippurate, a marker of polyphenol metabolism, or shifts in acylcarnitines indicating more fat oxidation) can validate the engagement of desired metabolic pathways. Metabolomics can also detect production of active cinnamon metabolites (such as cinnamic acid, phase II conjugates, etc.) and correlate them with physiological outcomes. This helps in understanding dose–response and the time-course of cinnamon’s effects. Furthermore, metabolomics in patients could reveal biomarkers of response for example, an individual whose metabolomic profile shows a strong shift in oxidative metabolites might be a “high responder” to cinnamon.

Nutrigenomic refers to how nutrients affect gene expression. Cinnamon’s components likely influence gene expression networks (e.g., upregulating antioxidant enzymes, downregulating inflammatory genes). Techniques like RNA sequencing of tissues or cells treated with cinnamon can identify transcriptional changes. One could imagine an experiment where muscle or adipocytes are incubated with cinnamaldehyde or cinnamon extract and then their gene expression is compared to control this might highlight pathways like PPAR signaling, stress response, or insulin signaling changes. Early studies have shown that cinnamon regulates genes for glucose transporter (GLUT4) and glycogenic enzymes in diabetic models [19,106,107]. Broadly, nutrigenomic data can clarify how cinnamon contributes to metabolic reprogramming on a gene level, and might identify new target genes or pathways (for example, does cinnamon activate genes related to browning of fat? Does it suppress lipogenesis genes in liver?).

Network pharmacology models how a compound (or mixture like cinnamon) interacts with multiple targets and how those interactions propagate through molecular networks. By integrating known protein targets of cinnamon compounds (from databases or lab assays) with metabolic disease networks, one can predict synergy and emergent effects. A network analysis might confirm that cinnamaldehyde targets TRPA1 and GPR109A, procyanidins target insulin receptor and PTP1B (just hypothetical), etc., and then show that collectively these targets modulate an insulin resistance network in a reinforcing way. Network pharmacology could also be used to design combination therapies, for example, combining cinnamon with another plant extract that hits complementary nodes (like fenugreek for AMPK, etc.) to cover more of the metabolic network [108,109]. Such in silico modeling, paired with experimental validation, accelerates discovery of effective nutraceutical combinations.

Using libraries of receptors or ion channels, scientists can profile which ones cinnamon’s constituents bind to or activate. Given the complexity of cinnamon, this is a big task, but miniaturized assays could test dozens of GPCRs or ion channels against cinnamon extract. This may reveal new targets. Perhaps a less-studied GPCR that influences metabolism is activated by a cinnamon compound, opening up a new avenue for research. Conversely, CRISPR-based functional genomics in cell models (knocking out certain receptors or channels) could pinpoint which are essential for cinnamon’s action (e.g., if removing TRPA1 in intestinal cells abrogates GLP-1 release with cinnamon, it confirms that target).

In essence, omics technologies will allow a more precise mapping of cinnamon’s molecular footprint in the body, helping to transform what is currently an empiric nutraceutical practice into a more predictable, quantified intervention. This precision will aid regulatory acceptance as well, by providing mechanistic and safety data at a granular level.

### 9.2. Personalized Nutrition for Metabolic Health

Just as not all drugs work uniformly for all patients, nutraceuticals like cinnamon may have variable efficacy depending on individual factors. The concept of personalized nutrition aims to tailor dietary interventions to an individual’s genetic makeup, microbiome, lifestyle, and metabolic status.

Polymorphisms in genes encoding cinnamon’s targets (ion channels, GPCRs, metabolic enzymes) could influence response. For example, variants in TRPA1 gene might alter channel sensitivity to cinnamaldehyde some people could be “high responders” feeling the burn and reaping metabolic effects, while others with a less sensitive TRPA1 might respond less. Similarly, genetic differences in the enzyme CYP2A6, which converts coumarin to 7-hydroxycoumarin, affect coumarin’s hepatotoxic risk slow metabolizers are at higher risk of liver damage from coumarin [27]. A future scenario might involve genotyping individuals: those with risk genotypes for coumarin toxicity would strictly use Ceylon cinnamon; those with certain GPCR polymorphisms that make them less responsive might need higher doses or different nutraceuticals. There is also the angle of taste receptor genetics: some people’s taste GPCRs may respond differently to cinnamon’s flavor compounds, potentially affecting cephalic-phase insulin release or gut–brain signaling.

The gut microbiota is crucial in metabolizing polyphenols. An individual’s microbiome composition can dictate how much of cinnamon’s polyphenols are converted into active metabolites. For instance, if someone lacks the bacterial strains that convert cinnamate to phenylpropionate (just hypothetical), they might not benefit from that pathway. Conversely, cinnamon could shape the microbiome in ways beneficial for metabolism studies have noted increases in probiotic strains and decreases in pathobionts with cinnamon supplementation [102]. Personalized nutrition could mean adjusting cinnamon dose based on microbiome profiles, or co-supplementing with probiotics that enhance cinnamon compound biotransformation. For example, if a certain Bifidobacterium helps break down polyphenols into insulin-sensitizing compounds, ensuring its presence might boost cinnamon’s efficacy. Metagenomic analysis of stool from responders vs. non-responders to cinnamon could identify microbial signatures associated with success.

People with different subtypes of diabetes or obesity might respond differently. For a lean diabetic with more of an insulin secretion defect, cinnamon’s GLP-1/insulinotropic effects may be particularly useful. In an obese, insulin-resistant person, the adipocyte-targeted effects (inflammation reduction, adiponectin increase) might be more pertinent. Thus, tailoring use perhaps dose or extract type to the phenotype could optimize results. Precision might also involve combining cinnamon with other nutraceuticals in a personalized mix. For instance, if one has high postprandial glucose but low fasting focus on cinnamon for post-meal effects; if another has fatty liver issues, maybe combine cinnamon with berberine or milk thistle for liver-targeted synergy.

Personalization also means accounting for diet and activity. Cinnamon might work best in the context of a diet that provides the right substrates for it to act (for example, enough protein to see an effect on incretins, or not too high alcohol which might stress the liver when combined with coumarin). Timing relative to exercise or time of day (maybe cinnamon in the evening could reduce morning fasting glucose by curbing overnight hepatic glucose output via its continuing effects as one study indicated cinnamon taken at dinner improved next morning glucose) [6]. Digital health tools and continuous glucose monitors (CGMs) now allow individuals to test how cinnamon affects their glucose in real time. Already, some CGM-wearing folks experiment with spices; aggregated data could guide who benefits most perhaps those with higher glycemic variability see more stabilization with cinnamon.

As mentioned, genotypes for coumarin metabolism or liver sensitivity can dictate safe use. Also, individuals on multiple medications need personalized advice (e.g., cinnamon might have mild anticoagulant properties as it can inhibit platelet aggregation in vitro due to cinnamaldehyde’s effects so someone on warfarin might need caution with very high doses) [27]. If a patient is on a β-blocker, the β-adrenergic activation by cinnamon might be blunted; knowing that can manage expectations or suggest a different adjunct (or even the fact that β-blockers could reduce metabolic benefits by blocking those receptors an interesting consideration).

In summary, personalized nutrition will ensure cinnamon’s benefits are maximized and any risks minimized for each individual. By integrating personal data genes, microbiome, health status healthcare providers could recommend whether cinnamon is appropriate, in what form, and how much. This moves away from one-size-fits-all supplementation to a targeted strategy. Cinnamon, with its multi-target nature, might not be dramatically potent in all people across the board, but in the right person at the right dose it could be significantly beneficial. Future clinical trials may stratify participants by genotype or microbiome to see where effects are strongest, paving the way for precision use.

## 10. Conclusions

Cinnamon, far from being just a common kitchen spice, emerges as a compelling membrane-active metabolic nutraceutical. Through a constellation of phytonutrients—cinnamaldehyde, eugenol, polyphenols, and others—cinnamon influences fundamental cell-signaling mechanisms in metabolic tissues. It modulates ion channels and GPCRs on the surfaces of cells, thereby orchestrating a host of beneficial effects: enhanced insulin secretion, improved insulin sensitivity, appetite regulation, increased energy expenditure, and reduced inflammatory stress. This review has highlighted how cinnamon’s components act as multi-target modulators, engaging targets such as TRP ion channels (TRPA1, TRPV1) and key GPCRs (adrenergic receptors, incretin receptors, GPR109A, etc.) to re-tune the complex signaling networks that go awry in metabolic diseases.

By targeting ion channels and GPCRs, the integrative “hubs” of cell signaling, cinnamon addresses metabolic dysfunction at a higher level of control compared to therapies aimed at single enzymes or end pathways. Ion channels provide immediate responses to metabolic stimuli (e.g., triggering insulin granule release via Ca^2+^ influx), while GPCRs coordinate longer-term and systemic responses (e.g., hormone release, gene expression changes). Cinnamon’s ability to impact both allows it to correct course on multiple fronts: not only does it lower blood sugar, but it also tackles underlying insulin resistance and even aspects of weight gain. We saw evidence from the last decade (2015–2025) that cinnamon supplementation can modestly lower HbA1c, fasting glucose, and improve the lipid profile in patients, and these clinical outcomes make sense in light of the molecular actions delineated, a testament to the translational significance of membrane signaling modulation.

Importantly, cinnamon’s safety and accessibility make it an attractive adjunct therapy. While not a replacement for first-line medications in diabetes or obesity, it offers a complementary mode of action that can enhance overall management. Its use is essentially a form of network pharmacology via diet, nudging a whole web of metabolic signals toward homeostasis. Ion channels and GPCRs, once underrated, are gaining recognition as viable intervention points in metabolic diseases, and cinnamon is a practical means to influence those targets concurrently and gently. It epitomizes a “food as medicine” approach that aligns with holistic management of chronic diseases.

That said, realizing the full potential of cinnamon in metabolic health will require continued research and careful application. Standardization of cinnamon extract, understanding individual variability (who responds best and why), and integrating cinnamon use with conventional treatments are the next steps. The future may see a precision nutrition paradigm where a person’s genetic and metabolic profile might indicate that they should use a cinnamon–coumarin-free extract to activate the TRPA1 and GPR109A pathways, which may be specifically beneficial for them.

In closing, the story of cinnamon in metabolic disease exemplifies a broader principle: targeting membrane signaling, the interfaces through which cells perceive and react to their environment, can yield broad and favorable outcomes in complex diseases. Ion channels and GPCRs serve as control knobs for metabolism, and cinnamon has shown us that a natural substance can turn multiple knobs in harmony. This not only improves metabolic parameters but does so in an “integrative” way, aligning with our body’s own regulatory logic. Embracing such strategies, alongside conventional therapies, could mark a shift toward more comprehensive and physiology-driven management of metabolic disorders. Cinnamon’s journey from spice rack to clinical study highlights the immense value that traditional nutraceuticals can offer when examined through the lens of modern science, a convergence of ancient wisdom and cutting-edge medicine that ultimately benefits patients with safer, multi-faceted interventions.

## Figures and Tables

**Table 1:** Key ion channels influenced by cinnamon phytonutrients and their metabolic roles.

Ion Channel	Expression/Role in Metabolism	Cinnamon Phytonutrient & Action	Metabolic Effect	Refs.
TRPA1 (Transient Receptor Potential Ankyrin 1)	Sensory neurons (pain fibers) are also found in intestinal enteroendocrine cells and pancreatic β-cells. Senses electrophiles, mediates neurogenic inflammation, and regulates hormone release (ghrelin, etc.).	Cinnamaldehyde Agonist (covalent activator of TRPA1 channels). Eugenol agonist at higher doses.	↑ Ca^2+^ influx and depolarization in TRPA^1+^ cells; induces insulin secretion from β-cells; in gut, ↓ ghrelin & ↑ GLP-1/CCK release (satiety signals); in sensory nerves, triggers reflex sympathetic activation for thermogenesis. Net effect: appetite suppression, improved glucose tolerance, enhanced energy expenditure, and weight reduction.	[7,19,31]
TRPV1 (Transient Receptor Potential Vanilloid 1)	Sensory neurons (heat/pain fibers) are expressed in the GI tract, skeletal muscle, and adipose tissue. Detects noxious heat and capsaicin. Activation leads to catecholamine release and enhanced metabolism.	Eugenol Partial agonist (vanilloid that can activate and subsequently desensitize TRPV1). (Capsaicin from chili prototypical agonist used as a reference for cinnamon’s effects.	Thermogenic and metabolic stimulation: Activation in gut nerves increases fat oxidation and energy expenditure (capsaicin-like effect). TRPV1 in muscle enhances oxidative fiber formation (↑ endurance metabolism). In adipose, TRPV1 activation reduces inflammation and promotes browning of fat (via SNS outflow). Cinnamon’s contribution is minor compared to capsaicin, but eugenol and related compounds add to warming and lipid oxidation effects.	[18,29,32,55]
Voltage-Gated Ca^2+^ Channels (L-type Ca^2+^ channels)	Pancreatic β-cells (trigger insulin release upon activation); vascular smooth muscle (controls contraction); muscle cells (excitation-contraction coupling).	Polyphenolic polymers Modulators (cinnamon extract may enhance β-cell Ca^2+^ currents or protect channel function). Possibly, cinnamaldehyde metabolite (cinnamic acid) acts as a mild Ca^2+^ channel antagonist in vessels.	In β-cells, facilitated insulin secretion, Ca^2+^ influx is required for exocytosis, and cinnamon-treated cells show improved insulin release. In vessels: vasodilation, cinnamon is linked to reduced calcium influx in VSMCs, aiding blood pressure control. Improved microcirculation and insulin delivery ensue. Overall, supports better glucose uptake and tissue perfusion.	[6,16]
K-ATP (ATP-sensitive K^+^ channel)	Pancreatic β-cells (set resting potential; closure triggers insulin release); hypothalamic neurons (glucose sensing).	*No direct agonist in cinnamon is known.* (Indirect: cinnamaldehyde’s TRPA1 activation bypasses K-ATP by depolarizing β-cells through Ca^2+^ influx).	Normally, K-ATP is the target of sulfonylureas for ↑ insulin. Cinnamon’s route: insulinotropic effect without inhibiting K-ATP, reducing the risk of over-secretagogue effect. Helps in conditions where K-ATP responsiveness is impaired (glucotoxic β-cells) by providing alternate depolarization.	[7]
Other TRP channels (TRPM8, TRPV2/3/4)	Adipose TRPV4 (regulates adipocyte inflammation & browning); TRPM8 (cold sensor, minimal metabolic role in cinnamon context). TRPV2/3 in pancreas and macrophages (possible roles in insulin secretion/inflammation).	Cinnamaldehyde has no direct effect on TRPM8 (cooling pathway). It possibly influences TRPV4 indirectly via reduced inflammation (cinnamon’s anti-inflammatory effects might relieve TRPV4-mediated inhibition of browning).	TRPV4: Cinnamon’s lowering of adipose inflammation may relieve TRPV4’s anti-browning effect, indirectly promoting healthier adipose function (TRPV4 inhibition is known to browning and improve insulin sensitivity). TRPM8: Not activated by cinnamon, hence not directly relevant; however, a combination of cooling (TRPM8) and cinnamon (TRPA1) could synergize in appetite control (conceptual).	[18,25]

## Data Availability

No data was produced from this study; all data is contained in this article and published paper in the references.
